# Editorial of Special Issue “Inflammasomes and Inflammation”

**DOI:** 10.3390/ijms23052489

**Published:** 2022-02-24

**Authors:** Young-Su Yi

**Affiliations:** Department of Life Sciences, Kyonggi University, Suwon 16227, Korea; ysyi@kgu.ac.kr; Tel.: +82-31-249-9644

Although inflammation is a host-protective mechanism from infection and cellular danger signals, chronic inflammation is a major risk factor for various human diseases [1,2,3]. Therefore, much effort has been made to understand the mechanisms of inflammatory responses and developing anti-inflammatory therapeutics. An inflammatory response consists of two successive steps; (1) “priming” which prepares inflammatory response by increasing the expression of inflammatory molecules and (2) “triggering” which activates inflammatory responses by inducing inflammasome activation. Previous studies have mainly focused on priming step, however, recent studies have demonstrated that the key feature of the triggering step is the activation of inflammasomes, which are intracellular protein complexes comprising pattern recognition receptors and inflammatory molecules [4,5,6,7,8,9], and also reported roles of inflammasomes in many human inflammatory diseases [4,5,6,7,8,9,10,11], providing strong evidence for new potential targets for novel anti-inflammatory drug development. However, inflammasome functions and their dysregulation in human inflammatory diseases still need to be investigated.

This Special Issue invited original research, reviews, and perspectives with a focus on, but not limited to, the mechanisms of inflammasome regulation, the role of inflammasomes in inflammatory responses and disease, the identification and validation of novel molecules regulating inflammasome functions, and potential inflammasome-targeted therapeutics.

The research article by Hytti et al. analyzed the effect of dysfunctional protein clearance on inflammation and inflammasome activation in induced pluripotent stem-cell-derived retinal pigment epithelial (iPSC-RPE) cells generated from an age-related macular degeneration (AMD) patient. This study demonstrated that AMD patient-derived iPSC-RPE cells show signs of a disease-specific phenotype, including a chronic low-level inflammasome activation and proteostasis dysregulation, which possibly suggests the appearance of drusen in the retina of AMD patients [12].

The research article by Park et al., demonstrated the roles of soluble endoglin (sENG) and vascular endothelial growth factor (VEGF)-A overexpression induced dysplastic vessel formation and reported that sENG provokes microglia to express angiogenic (VEGF-A, Notch-1, TGF-b) and inflammatory molecules (NLRP3 inflammasome components–NLRP3, ASC, and caspase-1 and pro-inflammatory cytokines–IL-1b, TNF-a, and IL-6) which are possibly involved in EC dysfunction, suggesting the contribution of microglia to the pathology of sENG-associated vascular malformations via modulating angiogenic and inflammatory responses [13].

The final research article by Jang et al. investigated the renoprotective effect of endothelial progenitor cells (EPCs) in ischemia-reperfusion injury (IRI), a major cause of acute kidney injury (AKI) and progression to chronic kidney disease (CKD) using an IRI mouse model. This study demonstrated that inflammasome-mediated inflammation accompanied by immune modulation and fibrosis is a potential target of EPCs as a treatment for IRI-induced AKI and the prevention of progression to CKD [14].

The review article by Péladeau and Sandhu summarized recent studies investigating the regulatory role of NLRP3 inflammasome in neuromuscular diseases that affect the peripheral nervous system and amyotrophic lateral sclerosis, which affects the central nervous system, which provides insight into the potential therapeutic strategy to target the NLRP3 inflammasome components for alleviating the detrimental phenotype of neuromuscular diseases and improving clinical outcomes [15].

The review article by Yi highlights the studies investigating the functional cooperation between various inflammasomes and methyltransferases, especially, DNA methyltransferases (DNMTs) and histone methyltransferases (HMTs) in the regulation of inflammatory responses and diseases [16]. This review provides insight into the development of anti-inflammatory therapeutics that selectively target inflammasomes and methyltransferases [16].

The review article by Cheon and Koo discussed the current literature regarding the inflammatory responses induced by the functional interaction between inflammasomes and the severe acute respiratory syndrome coronavirus 2 (SARS-CoV-2) in the coronavirus disease 2019 (COVID-19) patients and suggests that the inflammasome and its regulation have the potential as therapeutic targets for patients with COVID-19 [17].

The final review article by Akther et al. discussed the current studies demonstrating the role of post-translational modification (PTM) of inflammasomes in inflammasome-induced inflammatory responses and disorders. This review focused on the regulation of NLRP3 ubiquitination in NLRP3 inflammasome-activated inflammatory responses and disorders and provides insight into the NLRP3 ubiquitination modulators as potential therapeutic agents to regulate the activation of NLRP3 inflammasomes and to prevent and treat inflammatory disorders [18].

Overall, the topic of this special issue highlights the regulatory roles of inflammasomes in inflammatory responses and diseases and the underlying molecular and cellular mechanisms. We hope this special issue attracts the attention and interest of the scientific community to further contribute to future research demonstrating new mechanisms of inflammasomes in inflammatory responses and human diseases and sheds light on the development of novel therapeutics to prevent and treat various human diseases via targeting inflammasomes.

## References

[1] Gilroy D.W. (2021). Resolving inflammation. Nat. Rev. Immunol..

[2] (2017). A current view on inflammation. Nat. Immunol..

[3] Furman D., Campisi J., Verdin E., Carrera-Bastos P., Targ S., Franceschi C., Ferrucci L., Gilroy D.W., Fasano A., Miller G.W. (2019). Chronic inflammation in the etiology of disease across the life span. Nat. Med..

[4] Mathur A., Hayward J.A., Man S.M. (2018). Molecular mechanisms of inflammasome signaling. J. Leukoc. Biol..

[5] Christgen S., Place D.E., Kanneganti T.D. (2020). Toward targeting inflammasomes: Insights into their regulation and activation. Cell Res..

[6] Yi Y.S. (2017). Caspase-11 non-canonical inflammasome: A critical sensor of intracellular lipopolysaccharide in macrophage-mediated inflammatory responses. Immunology.

[7] Matikainen S., Nyman T.A., Cypryk W. (2020). Function and Regulation of Noncanonical Caspase-4/5/11 Inflammasome. J. Immunol..

[8] Broz P., Dixit V.M. (2016). Inflammasomes: Mechanism of assembly, regulation and signalling. Nat. Rev. Immunol..

[9] Rathinam V.A.K., Zhao Y., Shao F. (2019). Innate immunity to intracellular LPS. Nat. Immunol..

[10] Fernandes F.P., Leal V.N.C., Souza de Lima D., Reis E.C., Pontillo A. (2020). Inflammasome genetics and complex diseases: A comprehensive review. Eur. J. Hum. Genet. EJHG.

[11] Li Y., Huang H., Liu B., Zhang Y., Pan X., Yu X.Y., Shen Z., Song Y.H. (2021). Inflammasomes as therapeutic targets in human diseases. Signal Transduct. Target. Ther..

[12] Hytti M., Korhonen E., Hongisto H., Kaarniranta K., Skottman H., Kauppinen A. (2021). Differential Expression of Inflammasome-Related Genes in Induced Pluripotent Stem-Cell-Derived Retinal Pigment Epithelial Cells with or without History of Age-Related Macular Degeneration. Int. J. Mol. Sci..

[13] Park E.S., Kim S., Yao D.C., Savarraj J.P.J., Choi H.A., Chen P.R., Kim E. (2022). Soluble Endoglin Stimulates Inflammatory and Angiogenic Responses in Microglia That Are Associated with Endothelial Dysfunction. Int. J. Mol. Sci..

[14] Jang H.N., Kim J.H., Jung M.H., Tak T., Jung J.H., Lee S., Jung S., Chang S.H., Kim H.J. (2022). Human Endothelial Progenitor Cells Protect the Kidney against Ischemia-Reperfusion Injury via the NLRP3 Inflammasome in Mice. Int. J. Mol. Sci..

[15] Peladeau C., Sandhu J.K. (2021). Aberrant NLRP3 Inflammasome Activation Ignites the Fire of Inflammation in Neuromuscular Diseases. Int. J. Mol. Sci..

[16] Yi Y.S. (2021). Functional Interplay between Methyltransferases and Inflammasomes in Inflammatory Responses and Diseases. Int. J. Mol. Sci..

[17] Cheon S.Y., Koo B.N. (2021). Inflammatory Response in COVID-19 Patients Resulting from the Interaction of the Inflammasome and SARS-CoV-2. Int. J. Mol. Sci..

[18] Akther M., Haque M.E., Park J., Kang T.B., Lee K.H. (2021). NLRP3 Ubiquitination-A New Approach to Target NLRP3 Inflammasome Activation. Int. J. Mol. Sci..

